# A Dynamic Model for Estimating the Interaction of ROS–PUFA–Antioxidants in Rabbit

**DOI:** 10.3390/antiox11030531

**Published:** 2022-03-10

**Authors:** Simona Mattioli, Corrado Dimauro, Alberto Cesarani, Alessandro Dal Bosco, Desiree Bartolini, Francesco Galli, Anna Migni, Bartolomeo Sebastiani, Cinzia Signorini, Camille Oger, Giulia Collodel, Cesare Castellini

**Affiliations:** 1 Department of Agricultural, Environmental and Food Science, University of Perugia, Borgo 20 Giugno, 74, 06123 Perugia, Italy; alessandro.dalbosco@unipg.it (A.D.B.); cesare.castellini@unipg.it (C.C.); 2 Department of Agricultural Sciences, University of Sassari, Sassari, Viale Italia, 39, 07100 Sassari, Italy; dimauro@uniss.it (C.D.); acesarani@uniss.it (A.C.); 3 Department of Pharmaceutical Sciences, University of Perugia, Via Enrico Dal Pozzo, 06126 Perugia, Italy; desirex85@hotmail.it (D.B.); francesco.galli@unipg.it (F.G.); 4 Department of Life Science and System Biology, Università di Torino, Via Accademia Albertina, 13, 10123 Torino, Italy; anna.migni@edu.unito.it; 5 Department of Chemistry, Biology and Biotechnology, Via del Giochetto, University of Perugia, 06126 Perugia, Italy; bartolomeo.sebastiani@unipg.it; 6 Department of Molecular and Developmental Medicine, University of Siena, Policlinico Santa Maria alle Scotte, Viale Bracci 16, 53100 Siena, Italy; cinzia.signorini@unisi.it (C.S.); giulia.collodel@unisi.it (G.C.); 7 Institut des Biomolécules Max Mousseron (IBMM), Pôle Chimie Balard Recherche, 1919 route de Mende, CEDEX 05, 34293 Montpellier, France; camille.oger@umontpellier.fr

**Keywords:** metabolism, dynamic model, oxidation, antioxidants, polyunsaturated fatty acids

## Abstract

Defining optimal nutrition in animals and humans remains a main scientific challenge. The objective of the work was to develop a dynamic model of reactive oxygen species (ROS)–polyunsaturated fatty acid (PUFA)–antioxidant homeostasis using the rabbit as a model. The problem entity was to evaluate the main metabolites generated from interactions between traits included in the conceptual model and identified by three main sub–models: (i) ROS generation, (ii) PUFA oxidation and (iii) antioxidant defence. A mathematical model (VENSIM software) that consisted of molecular stocks (INPUTs, OUTPUTs), exchange flows (intermediate OUTPUTs) and process rates was developed. The calibration was performed by using standard experimental data (Experiment 1), whereas the validation was carried out in Experiments 2 and 3 by using supra–nutritional dietary inputs (VIT E+ and PUFA+). The accuracy of the models was measured using 95% confidence intervals. Analytical OUTPUTs (ROS, PUFA, Vit E, Ascorbic acid, Iso–/NeuroProstanes, Aldehydes) were well described by the standard model. There was also good accuracy for the VIT E+ scenario, whereas some compensatory rates (K*c1*–K*c4*) were added to assess body compensation when high levels of dietary PUFA were administered (Experiment 3). In conclusion, the model can be very useful for predicting the effects of dietary treatments on the redox homeostasis of rabbits.

## 1. Introduction

Reactive oxygen species (ROS) such as peroxides, the superoxide anion, the hydroxyl radical and singlet oxygen have complex interactions with the different classes of biomolecules and physiological processes of living organisms [1], thus playing fundamental roles in health and disease [2].

In isolated mitochondria, it is estimated that 0.1–2% of oxygen consumed produces ROS [3]. Depending on their relative concentrations and site of production, these molecular species can damage different cellular targets, including proteins [4], nucleic acids [5] and lipids [6]. As a consequence, these reactive species are reported to contribute to ageing processes [7] and age–related diseases (such as neurodegeneration, cancer and cardiovascular disease) [8]. ROS are also a crucial factor in male infertility because they could lead to sperm dysfunction [9].

Organisms have evolved molecular systems, including enzymes (metalloproteins such as superoxide dismutase and catalase [10], as well as thiol–based redox couples) [11] and non–enzymatic substances (i.e., glutathione, tocols, ascorbic acid (AA) and carotenoids) [12], to scavenge ROS or to react with them in order to maintain their physiological effects [8]. In fact, controlled generation of ROS, particularly hydrogen peroxide (H_2_O_2_), modulates virtually all cellular processes and is essential for living systems. A prime example is the presence of ROS in human/animal sperm, which activates spermatozoa capacitation to trigger egg fertilisation [13,14]. Again, H_2_O_2_ and other ROS play essential functions in biological signalling pathways spanning growth [15], cell differentiation [16] and migration [17] and immune system function [18]. However, if ROS production exceeds the physiological needs and the detoxification capacity, a cascade of negative responses and inflammation mechanisms can develop [19] and compensatory mechanisms occur at the transcriptional level to repair ROS–induced damage [20].

Lipid oxidation (peroxidation) is a major occurrence in both the toxic and physiological functions of cellular ROS. Increased lipid peroxidation may modify the structure and function of several classes of lipids, especially polyunsaturated fatty acids (PUFAs), including those esterified to triacylglycerols or membrane phospholipids of tissues, and free fatty acids (FFAs) [21]. Double bonds in the carbon chain of PUFAs are a main target of the electrophilic properties of ROS. Modifications mainly occur in n–6 and n–3 long–chain PUFAs (LC–PUFAs), such as alpha–linolenic acid (ALA: 18:3 n–3), arachidonic acid (ARA: 20:4 n–6), eicosapentaenoic acid (EPA: 20:5 n–3) and docosahexaenoic acid (DHA: 22:6 n–3) [22,23]. This uncontrolled oxidation of membrane PUFAs may have many metabolic and cellular effects, such as structural and functional modifications of the lipid bilayer and the production of metabolites such as malondialdehyde (MDA), alkenals and isoprostanoids, which can also be biomarkers of oxidative stability and lipid mediators.

Such oxidative susceptibility holds great importance in the reproductive system, because LC–PUFAs, mainly docosapentaenoic acid (DPA) and DHA, constitute > 60% of the sperm membrane [24]. Furthermore, in male rabbits fed a diet rich in PUFAs, there are increased LC–PUFA concentrations in the sperm and testes, which positively affects sperm kinetics [14]. However, these positive effects are only found when the animals have a concomitant increase in antioxidant intake (vitamin E, vitamin C and/or Selenium) to counteract MDA generation [12,24,25].

MDA is an aldehyde that originates from the fragmentation of oxidised lipids. Conversely, isoprostanoids, which are mainly prostaglandin (PG)–like substances, are produced by free radical–induced autoxidation of PUFAs [6,26] and represent a very specific and sensitive index of lipid peroxidation. Isoprostanoids are derived from fatty acids (FAs) esterified from membrane phospholipids and are released in free form in biological fluids. The main isoprostanoids are F_2_–isoprostanes (F_2_–IsoPs), F_3_–IsoPs and F_4_–neuroprostanes (F_4_–NeuroPs), which originate from AA, EPA and DHA, respectively [27]. Dietary levels of n–3 PUFA that are too high can increase F_4_–NeuroPs and negatively affect the oxidative status of rabbit sperm [14]. However, at certain levels, F_4_–NeuroPs (7 ng/mL) can also have positive effects on human reproduction, increasing the number of sperm able to fertilise eggs [28].

Vitamin E (tocotrienols and tocopherols) is one of the most important fat–soluble dietary antioxidants, with alpha–tocopherol as the most active and abundant form found in animal tissues (as reviewed recently [29,30]). Vitamin E plays a main role in PUFA protection; it works synergistically with other co–antioxidants to scavenge the lipoperoxyl radicals (LOO^•^) produced in the lipid peroxidation chain reaction. In a sequential process, two molecules of LOO^•^ generate a tocopheroxyl radical intermediate and then a relatively stable oxidation metabolite, namely tocopherylquinone (TQ) [20,31]. When the primary defences are not sufficient, other mechanisms, such as transcription of genes and transduction of essential proteins, interfere with the oxidative response (i.e., the cytochrome P450 [CYP450] family [32]). Furthermore, the tocopheroxyl radical can be reduced back to tocopherol by the activity of a redox antioxidant system that includes AA and thiol–dependent enzymes (e.g., glutathione reductase) as the main players [12], and the scavenging function of vitamin E (Vit E) cooperates with that of the membrane peroxidase, especially phospholipid hydroperoxide 4 (GPx4) [33].

Quantifying ROS, and, in general, the oxidation products of lipids and other biomolecules, remain very challenging. Current analytical tools provide different opportunities, but most of these exhibit a lack of specificity and sensitivity. Most common measurements are based on oxidation biomarkers, including aldehydes and isoprostanoids [34,35,36]. However, using these biomarkers without evaluating the molecules involved in the oxidative processes of tissues provides little information. Moreover, they do not represent the entire metabolic system, but only a portion of the mechanism.

The objective of this work was to build a model to simulate redox homeostasis of the rabbit in which the dynamic interactions of blood plasma indicators of ROS production and variations in PUFA and antioxidant levels (i.e., Vit E and AA) are considered during the response to metabolic and environmental stimuli (such as dietary intake of Vit E and PUFAs).

## 2. Materials and Methods

### 2.1. Cognitive Map Used for Definition, Calibration and Validation of the Model

The method reported by Sargent [37] was applied to build the model. Accordingly, we defined the Problem Entity, as well as the Conceptual and the Structural (i.e., computerised) models (Section 2.1.1, Section 2.1.2 and Section 2.1.3), integrating it with the states of Trucano et al. [38] and Dimokas et al. [39] to define the verification and validation procedures (Section 2.2).

Once we verified that the Structural model represents the Conceptual Description, we calibrated the model (showing the degree to which the model accurately fit the in vivo values (experiment 1, see Section 2.3) and successively validated it with another set of data (experiments 2 and 3, see Section 2.3).

#### 2.1.1. Problem Entity

As introduced earlier, evaluation of single products generated during the redox homeostasis of a system (i.e., aldehydes, isoprostanoids and antioxidants) from the interaction of multiple factors (ROS–PUFA–antioxidants) cannot describe the complexity and dynamic interactions of internal and external factors.

#### 2.1.2. Conceptual Model

**Figure 1:**
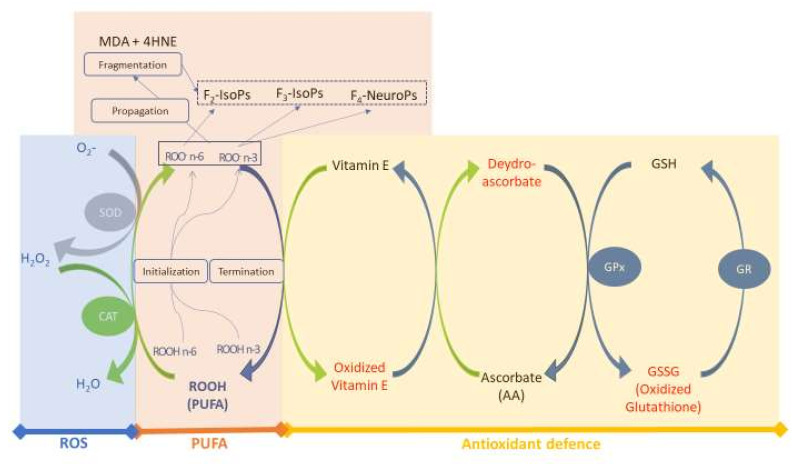
Conceptual model of the redox homeostasis system. Three sub–models are considered: the blue block includes ROS and enzyme antioxidants (SOD and CAT); the orange block includes PUFA and oxidised lipid forms (aldehydes [MDA and 4HNE] and isoprostanoids [F_2_–IsoPs, F_3_–IsoPs and F_4_–NeuroPs]); and the yellow block includes antioxidant defence (Vit E, AA and enzyme patterns, both in oxidised [red] and reduced [blue] forms). Abbreviations: MDA, malondialdehyde; 4HNE, 4–hydroxynonenal; GSH, glutathione; GPX, glutathione peroxidase; GR, glutathione reductase; F_2_–_3_–IsoPs, F_2_–_3_–isoprostanes; F_4_–NeuroPs, F_4_–Neuroprostanes; SOD, superoxide dismutase; CAT, catalase; ROS, reactive oxygen species. Modified from Mattioli et al. [12].

Figure 1 shows the map of the conceptual model used in this study. We considered three main sub–models for simulations and validation of the main biological interactions in the redox–homeostasis system of the rabbit plasma: (i) ROS generation, (ii) PUFA oxidation and (iii) antioxidant defences. The rate of every reaction depends on the metabolic balance of the model (see Section 2.1.3).

#### 2.1.3. Structural Model

**Figure 2:**
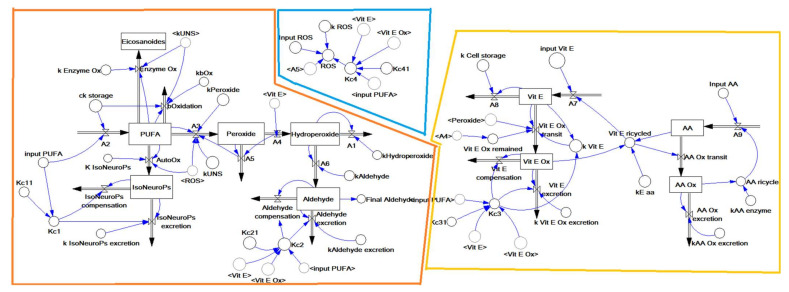
The structure of the dynamic model built with the VENSIM software. The model is split into three main sub–models: ROS generation (blue), PUFA oxidation (orange) and antioxidant defence (yellow) (as detailed in Figure 1). The variables inside the angle brackets (<>) are reported as a shadow of the stock variables. Abbreviations: AA, ascorbic acid; AA ox, oxidised ascorbic acid; PUFA, polyunsaturated fatty acids; ROS, reactive oxygen species; Vit E, vitamin E; Vit E ox, oxidised vitamin E.

**Figure 3:**
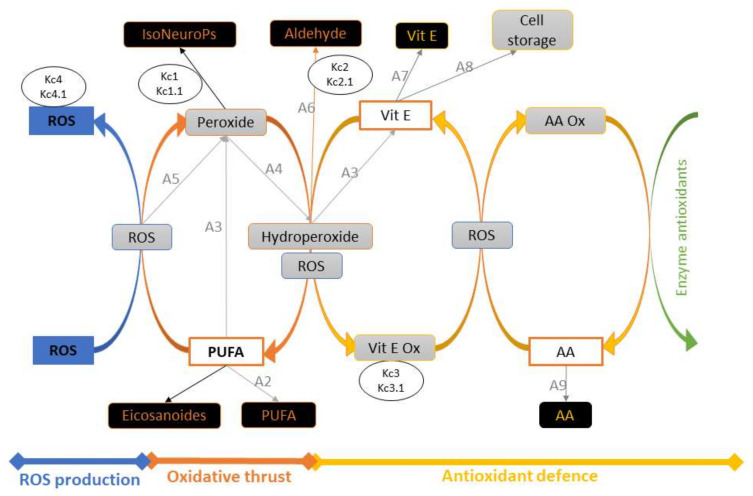
Schematic representation of the model, with INPUTs and OUTPUTs in white and black boxes, respectively. Intermediate OUPUTs are shown in grey boxes and result from molecular components (substrates and products) and sub–model interactions. The symbols *A1–A9* represent the metabolic pathways generating intermediate outcomes (see Section 2.1.3), and K*c1–4* and K*c1.1–4.1* are compensatory rates introduced after model verification by in vivo experimental data (see Section 2.2). Abbreviations: AA, ascorbic acid; AA ox, oxidised ascorbic acid; PUFA, polyunsaturated fatty acids; ROS, reactive oxygen species; Vit E, vitamin E; Vit E ox, oxidised vitamin E.

Inflows and outflows represent ‘gaining’ and ‘losing’ molecules, respectively. The ‘dynamic’ flow of substrates and products of a reaction, including transient intermediates (i.e., hydroperoxides, peroxides, Vit E oxidation, etc.) is represented by the reaction rates as reported in the following sections. The time considered is 1 h (3600 s), which represents about one fifth of the time for standard feed digestion in the rabbit (4–6 h) [36]. To build the operation map in VENSIM software [40], different traits were analytically determined (Section 2.5) and classified as INPUTs and OUTPUTs (Figure 2). The model also calculates intermediate OUTPUTs resulting from the interaction of several components. The symbols *A1*–*A9* represent the metabolic pathways that generate intermediate outcomes described in detail in Section 2.1.3. K*c1–4* and K*c1.1*–*4.1* represent compensatory rates introduced in the system after model validation (Figure 3; see Section 2.2).

##### Sub–Model 1: ROS Production

For *ROS*, the physiological concentrations and basal rate of generation are described in the model system as *ROS input* and k*ROS*, respectively. These variables are assumed to represent both the standard metabolism of a living system (i.e., the respiratory process, movement and cell metabolism [16]) and the compensatory response to external stimuli (K*c4* and K*c4.1*; e.g., hyperventilation, nutrition, stress, environmental perturbation, etc.), which can increase and even exceed the antioxidant capacity of the system and lead to oxidative stress [41]. *ROS output* (Equation (1)) is the sum of *ROS input* and ROS generated through the neutralisation of *Aldehydes* (*A6*; Equation (11)). Furthermore, a numeric value (i.e., 400) has been added to convert the units of the considered variables to moles. *A1* (Equation (2)) represents the reactive molecules originated by the reaction steps of Vit E.

*ROS* = ((*inputROS* + *A5* × 400) × k*ROS*) × K*c4* (1)

*A1* = k*Hydroperoxide* × *Hydroperoxide* (2)

##### Sub–Model 2: Oxidative Thrust

In the model, *PUFA* represents the main substrate of oxidation. *PUFA* can be introduced with the diet (*input PUFA*) to follow different metabolic routes associated with specific metabolic pathways (*A1*–*A6*; Equations (3)–(12)).

The first pathway (*A2*; Equation (3)) represents PUFA storage in tissues, regulated by a continuous rate (ck*storage*) mainly dependent on PUFA intake:

*A2* = *inputPUFA* × 1/ck*sorage* (3)

The second pathway encompasses different routes involved in eicosanoid generation. Eicosanoids can form through different pathways by the activity of a series of oxidoreductases, including cyclooxygenases (COX), lipoxygenases (LOX) and CYP450 isoenzymes (*EnzymeOx*; Equation (4)). COX produce prostaglandins (PGs) and thromboxanes (TXs); LOX form leukotrienes (LTs) and lipoxins (LXs); and CYP450s form various epoxy, hydroxy and dihydroxy derivatives. All these pathways influence the flux of eicosanoid molecules (k*EnzymeOx*) and PUFAs (k*UNS*).

*EnzymeOx* = k*EnzymeOx* × k*UNS* (4)

The third pathway is β–oxidation (*βOxidation*; Equation (5)) [42] that, independently of *ROS* activity, promotes LC–PUFA catabolism (reported in the system as k*UNS*) to produce energy in the mitochondria with a rate (k*βOx*) affected by the energy balance of the different tissues, negatively correlating with storage of PUFA in cells [43].

*βOxidation* = *PUFA* × k*βOx* × k*UNS* × 1/ck*storage* (5)

The fourth route is autoxidation of *PUFA* to produce isoprostanoids (*IsoNeuroPs*). These are generated from LC–PUFA in cell membranes. After oxidation, they are cleaved by phospholipase and released in the interstitial fluids. Consequently, *IsoNeuroPs* are found in the plasma and can be eliminated through phospholipase A2 [44] with urine (*IsoNeuro excretion*; Equation (6)) at a specific excretion rate (k*IsoNeuro excretion*). Their concentrations and autoxidation (*AutoOx*; Equation (7)) are assumed to depend on PUFA precursors and/or ROS (k*IsoNeuroPs*). Compensations introduced for this pathway are K*c1* and K*c1.1* (see Section 2.2).

*IsoNeuroPs excretion* = IF(K*c1* = 0)THEN(k*IsoNeuroPs excretion* × *IsoNeuroPs*)ELSE(k*IsoNeuroPs excretion* × *IsoNeuroPs* × K*c1*) (6)

*AutoOx* = IF(*ROS* > 0)THEN(*PUFA* × *IsoNeuroPs*)ELSE(0) (7)

The fifth pathway is PUFA oxidation that correlates with the unsaturation level (k*UNS*) of FA, ROS and antioxidant activity [45]. PUFA oxidation produces *Peroxides* (initialisation step, *A3*; Equation (8)), a phenomenon that can be prevented, at least in part, by the H atom–donating activity of Vit E. This is also expected to influence *Hydroperoxide* levels, depending on the downstream activity of membrane peroxidases (primary metabolites resulting from termination activity, *A4*; Equation (9)). If not properly scavenged, lipoperoxyl radicals and lipoperoxides undergo extensive fragmentation to form meta–stable–molecules, such as *Aldehydes* (secondary metabolites, *A6*; Equation (11)). In particular, one molecule of hydroperoxide is broken down into two molecules of aldehydes (*final Aldehydes*) [46]. The *Aldehyde* generation depends on antioxidants (reported as k*Aldehydes*), and on molecules’ susceptibility to oxidation (k*Perox*, *A5*; Equation (10)). Finally, aldehydes can be excreted by urine (*Aldehyde excretion*), assuming that the rate of excretion is related to their concentrations (k*Aldehyde excretion*; Equation (12)). This rate can change in relation to the activation of compensatory activities aimed at reducing the damage to lipids and proteins (K*c2* and K*c2.1*; as reported in the following scenarios Section 2.3). It should be underlined that the concentrations of these latter molecules could be affected by mechanisms of re–absorption, transport and/or excretion that are difficult to estimate with our experimental plan.

*A3* = IF(*ROS* > 0)THEN((*PUFA* × kU*NS* × k*Perox*) + *A5*)ELSE(0) (8)

*A4* = IF(*vit E* > 0)THEN(*vit E* × 1000)ELSE(0) (9)

Note that 1000 is the scale factor to compare Vit E to peroxides.

*A5* = IF((*Peroxide*-*A4*) > 0)THEN(*Peroxide*)ELSE(0) (10)

*A6* = *Hydroperoxide* × k*Aldehyde* (11)

*Aldehyde excretion* = IF(K*c2* = 0)THEN(*Aldehyde* × k*Aldehyde excretion*)ELSE(*Aldehyde* × k*Aldehyde excretion* × K*c2*) (12)

##### Sub–Model 3: Antioxidant Defence

Antioxidants are substances that delay or prevent the oxidation of other molecules. In our model, *Vit E* is the main fat–soluble antioxidant capable of preventing PUFA peroxidation. It works both as a scavenger of peroxyl radicals and an inhibitor of oxidoreductases involved in the enzymatic oxidation of PUFA. The antioxidant activity of Vit E results in the formation of α–TQ (*Vit Eox* and *Vit Eox remained* or *Vit Eox transit*, *A7*; Equations (13) and (14), respectively) with a molar rate of 1/2 with respect to peroxyl radicals scavenged during the H atom transfer process. Vit E in the model changes according to different metabolic pathways (*A7*–*A9*) and together with AA is regulated by Equations (13)–(20).

*Vit Eox transit* = IF(*Vit E* > *Peroxide*)THEN((*Vit E*/2) × k*Vit E* + *Vit E remained*/2)ELSE(*Vit Eox remained*/2) (13)

*A7* = *Vit E recycled* + *input Vit E* (14)

The availability of Vit E is modulated by a series of exogenous and endogenous factors [47]. Under physiological conditions, the main exogenous factor is dietary intake (*Vit E input*) [12]. Furthermore, Vit E (*A8*; Equation (15)) stored in the cells or other depots (*cell storage*) and recovered by recycling tocopheryl radicals formed during lipoperoxyl radical scavenging (*Vit E recycling*; Equation (16)) is the endogenous counterpart acting on the inner pool of the vitamin. Indeed, *Vit E* is regenerated by the intervention of *AA*, which acts as a secondary antioxidant (*A9*; Equation (17)), with a specific rate indicated as k*Vit E_AA*.

Vit E not recycled after oxidation or in excess is assumed to be excreted in the urine or bile (*Vit Eox excretion*) with a concentration–dependent rate (k*Vit Eox excretion*; Equation (18)). Compensatory mechanisms considered in this respect are indicated as K*c3* and K*c3.1*, which include CYP450 detoxification and other excretion processes [48]).

*A8* = *Vit E* × k*Cell storage* (15)

*Vit E recycle* = IF(*AA* > 0)AND(*Vit Eox* > 0)THEN(*AA* × k*E_AA*)ELSE(0) (16)

*A9* = *AA recycle* + *input AA* (17)

*Vit Eox excretion* = IF(K*c3* = 0)THEN(k*Vit Eox excretion* × *Vit Eox*)ELSE(*kVit Eox excretion* × *Vit Eox* × K*c3*) (18)

The same recycling process is considered in the case of AA that is oxidised (*AAox*) according to *AAox transit*. Thus, it is restored back to the reduced form (*AA recycling*; Equation (19)) by trans–hydrogenases modulated by glutathione reductase (GR) [49] and specific ascorbate reductases [50], with a rate identified in the model as k*AA enzyme*. Similarly to Vit E, AA is modulated by dietary intake (*AA input*), *AAox excretion* (Equation (20)), the rate of excretion (k*AAox excretion*) and *AA recycle.*

*AA recycle* = *IF*(*AAox* > *0*)*THEN*(k*AA enzyme* × *AAox*)*ELSE*(*0*) (19)

*AAox excretion* = k*AAox excretion* × *AAox* (20)

### 2.2. Verification and Validation of the Model

**Figure 4:**
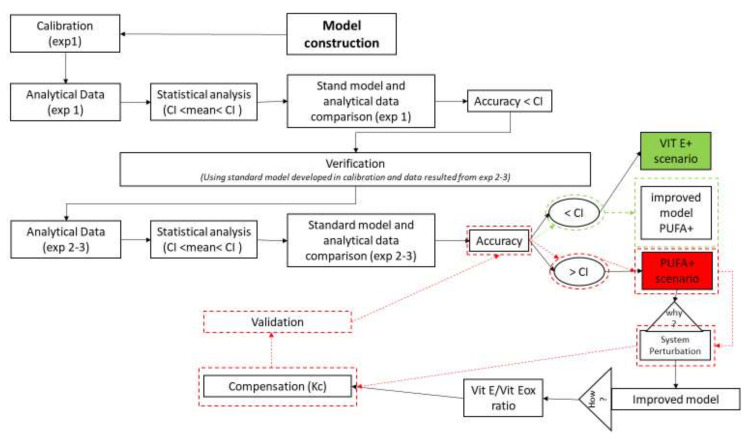
Cognitive map for calibration and validation of the model. The first step was to construct the model calibrated with analytical data (mean and 95% confidence interval [CI]) of Experiment 1 (exp 1). We considered the model to be accurate when the outcomes were included in the 95% CI. The second step was verification of this model by using data from Experiment 2 (exp 2, VIT E+) and Experiment 3 (exp 3, PUFA+). The VIT E+ scenario resulted in good accuracy (green box); thus, it did not require further adjustment. However, some OUTPUTs of the PUFA+ scenario were out of the 95% CI (red–coloured box) and showed unstable results. Hence, the model was improved through the introduction of compensatory values (K*c*) obtained from intermediate OUTPUTs (Vit E/Vit Eox ratio). After compensation, the system was validated again (boxes surrounded by red dashed lines indicate > CI, whereas boxes surrounded by green dashed lines indicate < CI).

We calibrated the model by using the data from Experiment 1 (standard model) to evaluate its accuracy. We validated the model by using data from Experiments 2 and 3, where the main INPUTs were changed (VIT E+ and PUFA+ scenarios; Figure 4); the fitness of the results was assessed.

We confirmed the model if the model outcomes of Experiments 2 and 3 were within the 95% CI (e.g., VIT E+). Otherwise, starting from the examination of final and intermediate OUTPUTs, we introduced compensation (K*c1*–k*c4* and K*c1.1*–K*c4.1*.; Equations (21)–(24); Section 3.2) to re–stabilise equilibrium and fitness and then generated new estimates and validated the model again (Figure 4).

K*c1* = IF(*input_PUFA* < 55.5)THEN(0)ELSE(K*c1.1*) (21)

K*c2* = IF(*input_PUFA* < 55.5)AND(*Vit E*/*Vit Eox* < 3.45)THEN(0)ELSE (K*c2.1*) (22)

K*c3* = IF(*input_PUFA* < 55.5)AND(*Vit E*/*Vit Eox* < 3.45)THEN(0)ELSE(K*c3.1*) (23)

K*c4* = IF(*input_PUFA* < 55.5)AND(*Vit E*/*Vit Eox* < 3.45)THEN(1)ELSE(K*c4.1*) (24)

We set the values (in moles) of PUFA <55.5 and the ratio Vit E/Vit Eox <3.45 based on the results obtained during validation of the model (Section 3.1). We subsequently used and compared data from animal experiments (Section 2.3).

### 2.3. Experimental Plan: Animals and Dietary Treatments

Thirty blood samples were used to test and validate the model in two experimental sessions. Experiment 1 tested and verified the standard model (*n* = 10 samples), whereas validation was performed by using the same number of samples in Experiments 2 and 3 (*n* = 10 samples for each experiment) and diets with supra–nutritional levels of Vit E and PUFA [51].

**Experiment 1.**

Samples were collected from 10 New Zealand White male rabbits, 140 days old, reared at an experimental facility of Siena University and fed *ad libitum* a standard diet (Table A1 of Appendix A) containing 50 mg/kg alpha–tocopheryl acetate (vitamin–mineral premix) [52].

**Experiments 2 and 3.**

We verified the model by using data from two distinct dietary interventions with high dosages of Vit E and PUFA:

○ High VITAMIN E (VIT E+): 10 rabbits of the same age and breed mentioned above were fed *ad libitum* the standard diet (50 mg/kg alpha–tocopheryl acetate by vitamin–mineral premix), including 200 mg/kg alpha–tocopheryl acetate.
○ High PUFA (PUFA+): 10 rabbits of the same age and breed mentioned above were fed *ad libitum* a standard diet supplemented with 10% extruded flaxseed and 200 mg/kg alpha–tocopheryl acetate, following the rabbit requirements for a flaxseed enriched diet [53]. This diet had a 1.34–fold higher concentration of ALA than the standard diet.

The dietary protocols involved 50 days of adaptation during which the rabbits were only monitored and a subsequent 60 days during which blood samples were collected every 2 weeks.

### 2.4. Samples Collection

About 2 mL of blood was taken from the auricular marginal vein, after the local application of an anaesthetic cream (EMLA^®^), using a 2.5 mL syringe fitted with a butterfly needle. Serum was obtained from blood samples coagulated at room temperature for 2 h, whereas plasma was obtained from blood samples collected in tubes containing Na_2_–EDTA (ethylenediaminetetraacetic acid) and centrifuged immediately at 2500× *g* for 15 min at 4 °C.

For the plasma F_2_–IsoP, F_3_–IsoP and F_4_–NeuroP determinations, butylhydroxytoluene (BHT) was added (90 µM, final concentration).

The experimental trial was conducted in accordance with the Guiding Principles in the Use of Animals and approved by the Animal Ethics Committee of the Siena University (CEL AOUS; authorisation n° 265/2018–PR, ISOPRO 7DF19.23).

### 2.5. Analytical Determinations

#### 2.5.1. Determination of the Fatty Acids

Lipids were extracted from the serum and feed according to the method of Folch et al. [54], and the esterification was carried out following the procedure of Christie [55].

The FA profile was determined by using a Varian gas chromatograph (CP–3800) equipped with a flame ionisation detector (FID) and a capillary column 100 m in length × 0.25 mm × 0.2 μm film (Supelco, Bellefonte, PA, USA). Helium was used as the carrier gas (0.6 mL/min). The split ratio was 1:20 for serum and 1:50 for feed. The oven temperature was programmed as reported by Mattioli et al. [56]. Individual fatty acid methyl esters (FAME) were identified by comparing the relative retention times of peaks in the sample with those of a standard mixture (FAME Mix Supelco; 4:0 to 24:0) plus cis–9, cis–12 C18:2; cis–9 cis–12 cis–15 C18:3; cis–9 cis–12 cis–15 C18:3 (all from Sigma–Aldrich, Steinheim am Albuch, Germany). The main PUFAs of both n–6 and n–3 series were quantified by using heneicosanoic acid methyl esters (Sigma Chemical Co., Schnelldorf, Germany) as an internal standard and are expressed in mg/mL of blood. Subsequently, each FA was converted to moles based on its specific molecular weight.

#### 2.5.2. Determination of Vit E and AA

Vit E, in both the reduced form (α–tocopherol [α–TOH]) and oxidised form (α–tocopherylquinone [α–TQ]), was quantified in plasma. Before lipid extraction, the samples were thawed at room temperature and homogenised by gentle pipetting.

α–TQ and α–TOH were quantified as described by Torquato et al. [20], with minor modifications. Briefly, 70 µL of plasma was spiked with α–TOH–d6 as an internal standard and mixed with 0.2 mL of sodium acetate buffer (pH 5) for the incubation (30 min at 37 °C) with 700 units of β–glucuronidase from *Helix pomatia* (Sigma–Aldrich, Steinheim am Albuch, Germany, G7017, Type HP–2). One hundred microlitres of glacial acetic acid and 1 mL of ethanol were added and, after mixing, the samples were incubated for 30 min at −20 °C and centrifugated at 3500 rpm for 5 min. Then, the ethanol was removed under a gentle stream of nitrogen and 2 mL of Milli–Q water was added to the sample. The lipid fraction was extracted twice by liquid/liquid partition with 3 mL of *n*–hexane/*n–tert* methyl tertiary–butyl ether (MTBE) mixture (2:1; *v/v*). After centrifugation at 3500 rpm for 5 min at 10 °C, the extracts were collected and dried under a stream of nitrogen. Then, the residues were resuspended with 80 µL of MOX reagent (Sigma Aldrich, TS–45950) and incubated at 70 °C for 2 h. The samples were further dried under nitrogen and then the analytes were derivatised with 50 µL of *N*–methyl–*N*–(trimethylsilyl)–trifluoroacetamide (Sigma–Aldrich, Steinheim am Albuch, Germany, 69479–5ML) in 50 µL of pyridine. Following incubation at 70 °C for 1 h, the samples were dried under a gentle stream of nitrogen and re–suspended in 125 µL of toluene for gas chromatography–mass spectrometry (GC–MS). Specifically, α–TOH and α–TQ were qualitatively and quantitatively assessed by using a mass spectrometer (Agilent Technologies, Milan, Italy; MSD 5975C, VLD with Triple Axis Detector) coupled to a gas chromatograph (GC 7890A) equipped with an Agilent VF–5 ms capillary column (15 m × 0.15 mm internal diameter [i.d.], 0.15 μm film thickness). The constant flow of the helium carrier gas was 1.0 mL/min and the injector operated in pulsed splitless mode (pressure 50 psi) at 300 °C. The oven temperature ramp for α–TOH and α–TQ was: from 120 °C (held for 1.5 min) to 240 °C at 113 °C/min, then to 270 °C at 8.5 °C/min (held for 1.5 min), to 350 °C at 28.3 °C/min, and finally the system was maintained under isothermal conditions for 5 min. The transfer line, ion source and quadrupole temperatures were set at 300, 230 and 150 °C, respectively. For each analyte and related recovery internal standard, the mass spectrometer operated in selected ion monitoring mode (EI+ SIM) at 70 eV: 508, 243 *m*/*z* for α–TOH–d6; 502, 237 and 430, 293 *m*/*z*, for α–TOH and α–TQ, respectively. We ensured high quality assurance and quality control of the performed methods by evaluating the testing linearity, detection limit, quantification limit, reproducibility and percentage of recovery values. The data are expressed as number of moles.

AA was measured in plasma by using a high–performance liquid chromatography system (HPLC) as described by Ross [57] with minor modifications as reported by Mattioli et al. [12]. AA was quantified by ultraviolet (UV) reverse–phase HPLC using a Waters 600 E System Controller HPLC equipped with a Waters Dual k 2487 detector (Milford, MA, USA) set at 262 nm. A 5 μm Ultrasphere ODS column (Beckman, San Ramon, CA, USA) was used with acetonitrile:water (49:51, *v/v*) as the mobile phase at a flow rate of 0.8 mL/min. AA concentrations were calculated by peak areas determined using an Agilent 3395 integrator (Agilent Technologies, Santa Clara, CA, USA); the results are expressed in moles.

#### 2.5.3. Determination of ROS, Malondialdehyde, F_2_–Isoprostane, F_3_–Isoprostane, and F_4_–Neuroprostane

ROS levels in plasma were evaluated by using a commercial kit (Diacron, Grosseto, Italy) and are expressed as moles of H_2_O_2_.

Lipid peroxidation in plasma was assessed by the MDA level using malondialdehyde tetrabutylammonium as a standard (Sigma–Aldrich, Steinheim am Albuch, Germany) and indicated in the model as Aldehydes. Rabbit plasma samples were mixed in a 0.04 M K^+^–phosphate buffer (pH 7.4) containing 0.01% BHT (1:5 *w/v*, 0 °C) to prevent artificial oxidation of free PUFA during the assay. The supernatants resulting from deproteinisation with acetonitrile (1:1) were used for aldehyde analysis after pre–column derivatisation with 2,4–dinitrophenylhydrazine [58]. The samples were stirred immediately, extracted with 5 mL of pentane and dried under a nitrogen flow. The aldehyde hydrazone was quantified by isocratic HPLC using a Waters 600 E system controller HPLC instrument equipped with a Waters Dual λ 2487 UV detector set at 307 nm. A 5 μm Ultrasphere ODS C18 column was used with a mobile phase composed of acetonitrile (45%) and 0.01 N HCl (55%) at a flow rate of 0.8 mL/min. The aldehyde concentration was calculated based on the peak areas using an Agilent 3395 integrator. The results are expressed as moles of Aldehyde.

Levels of isoprostanoids (free form plus esterified)—F_2_–IsoPs, F_3_–IsoPs and F_4_–NeuroPs—were determined by GC/negative–ion chemical ionisation tandem mass spectrometry (GC/NICI–MS/MS, Thermo Finnigan Instrument, Midland, Canada) as detailed by Mattioli et al. [12]. Prostaglandin F_2α_ (PGF_2α_–d_4_; 500 pg) was used as an internal standard. Solid phase extraction procedures were carried out according to a previous publication [59]. All final eluates were derivatised to convert the carboxylic group of isoprostanoids or PGF_2α_–d_4_ into pentafluorobenzyl ester and the hydroxyl group into trimethylsilyl ethers, as reported previously [26]. Derivatised isoprostanoids were detected and quantified by GC/NICI–MS/MS. The mass ions determined were the product ions at *m/z* 299 (F_2_–IsoPs), *m*/*z* 297 (F_3_–IsoPs) and *m*/*z* 323 (F_4_–NeuroPs). For the internal standard (PGF_2α_–d_4_), the mass ions determined were the product ions at *m*/*z* 303. All the product ions were derived from the [M–181]^−^ precursor ions. Reference molecules for F_2_–IsoPs, F_3_–IsoPs, and PGF_2α_–d_4_ were purchased (Cayman Chemical, Ann Arbor, MI, USA). 4(RS)–F_4t_–NeuroP, 10^®^−10–F_4t_–NeuroP and 10(S)−10–F_4t_–NeuroP were synthesised by C.O. and used as reference molecules for F_4_–NeuroP determination. The results are expressed in moles.

The concentration of all the blood traits was divided by 10^16^ to make the data easier to read.

### 2.6. Statistical Evaluation

A linear model (SAS, [60]) was used to evaluate the fixed effect of diet (Standard, VIT E+, PUFA+) on blood traits resulting from chemical evaluation. We used multiple comparisons (Bonferrini’s test) to establish the significance of differences between dietary treatments (*p* < 0.05). We calculated the mean, standard error and 95% CI of the analytical data.

VENISM software (release 9.1.0; Ventana Systems, UK Ltd., Wiltshire, UK) created several non–linear equations for estimating the outcomes at different lag–times based on the relationship between variables, which were imposed by the developer. We evaluated the accuracy of the models by comparing the 95% CI of the analytical data with the relative outputs estimated for calibration and then for the validation procedure.

As exemplified in the cognitive map (Figure 4), for Experiment 3 when the outcomes were far from the CI (e.g., PUFA+; Table 1), the model became unstable and needed compensation. We calculated this compensation from the effect of PUFA increase on the Vit E/Vit Eox ratio by fitting a polynomial regression curve. We used a first derivative equal to 0 to calculate the flex points (average, minimum and maximum) and we used the relative *y* value to assess the compensation rates (K*c1*–K*c4* and K*c1.1*–K*c4.1*) (Equations (21)–(24)). We re–evaluated the accuracy of the resulting model by using the above–mentioned procedure.

## 3. Results

### 3.1. Calibration of the Standard Model

**Figure 5:**
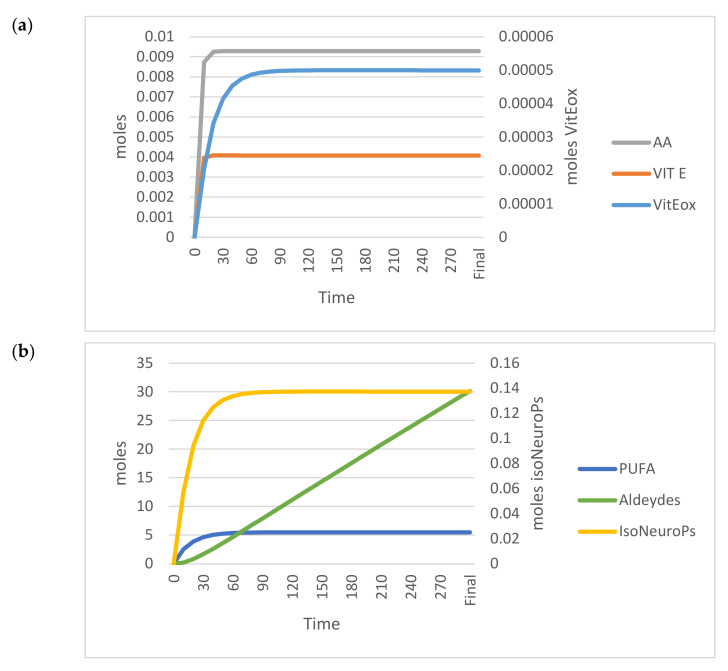
Time–dependent trend (300 min) of the main OUTPUTs obtained by applying the VENSIM software to the standard model. (**a**) AA, Vit E and Vit Eox; (**b**) PUFA, Aldehydes, IsoNeuroPs. Abbreviations: AA, ascorbic acid; IsoNeurops, Iso– and neuroprostanes; PUFA, polyunsaturated fatty acids; Vit E, vitamin E; Vit Eox, oxidised vitamin E.

Figure 5 shows the trends of the OUTPUTs analysed in the standard model.

Table 1 (standard model section) shows the analytical values and those estimated by the standard model (OUTPUTs). All estimated values for ROS, PUFA, Vit E, AA, IsoNeuroPs, Aldehydes and Vit Eox were within the 95% CI.

### 3.2. Model Validation in the VIT E+ and PUFA+ Scenarios

We compared the analytical and model outcomes for the VIT E+ and PUFA+ scenarios. As shown in the cognitive map (Figure 4), the VIT E+ scenario was estimated accurately with the standard model, whereas the PUFA+ scenario results were far from the 95% CI.

The VIT E+ scenario resulted in similar amounts of IsoNeuroPs and AA and lower Aldehydes than the standard one. The Vit E concentration resulting from addition to the diets was similar to what was recorded in the standard scenario (458.41 × 10^−5^ vs. 422.00 × 10^−5^ moles, respectively), but its oxidised form (Vit Eox) was 3–fold higher.

**Figure 6:**
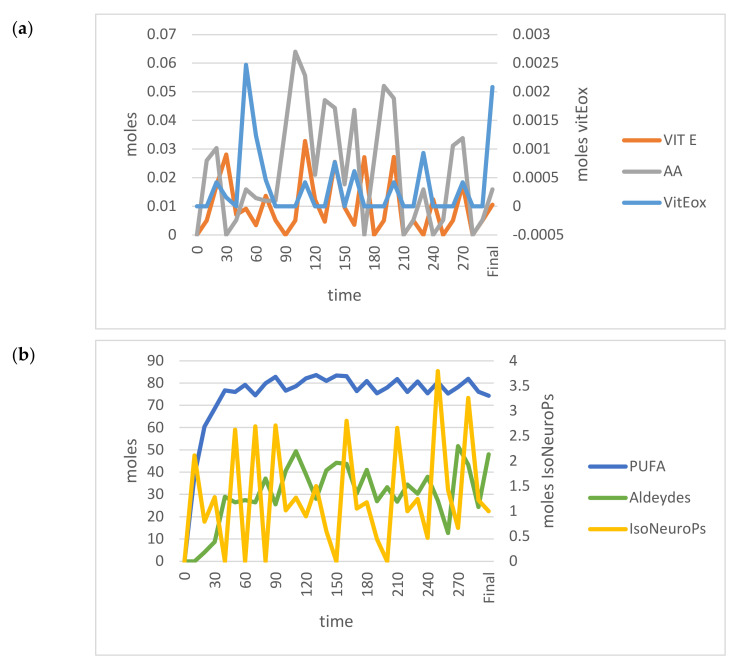
Time–dependent trends (300 min) of the main OUTPUTs obtained by applying the VENSIM software to the standard model when input PUFA increased (>55.5 moles). (**a**) AA, Vit E and Vit Eox; (**b**) PUFA, Aldehydes and IsoNeuroPs. Abbreviations: AA, ascorbic acid; IsoNeurops, Iso– and neuroprostanes; PUFA, polyunsaturated fatty acids; Vit E, vitamin E; Vit Eox, oxidised vitamin E.

**Figure 7:**
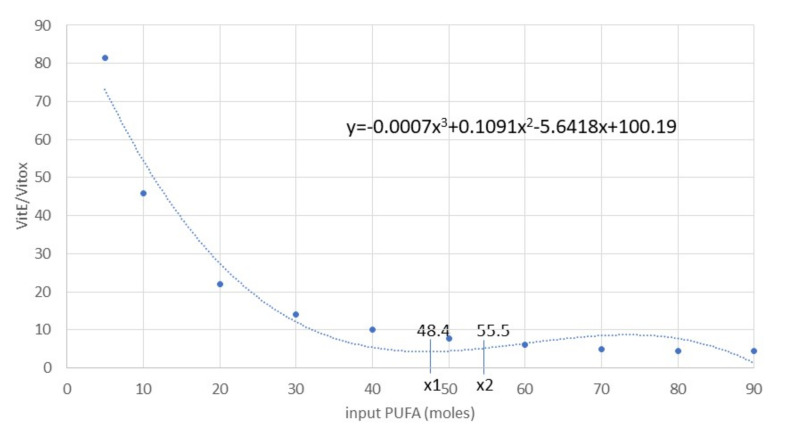
Polynomial regression curve of the Vit E/Vit Eox ratio and PUFA (R^2^ = 95%) data resulting from analytical determinations. The first derivative calculation produced the following flex points: x_average_ = 51.95, x_1_ = 48.41 (minimum), x_2_ = 55.48 (maximum). Abbreviations: PUFA, polyunsaturated fatty acids; Vit E, vitamin E; Vit Eox, oxidised vitamin E.

On the contrary, when PUFA INPUTs increased, the model outcomes became unstable (Figure 6) and far from the 95% CI (data not shown; IsoNeuroPs = 0.34 moles, Aldehydes = 16 moles, Vit Eox = 32.2 × 10^−5^ moles). In this situation, one of the most relevant disturbing factors was the Vit E/Vit Eox ratio, which decreased as PUFA increased. The flex points of this polynomial relationship correspond to about 55.5 moles PUFA and a Vit E/Vit Eox ratio of 3.45 (Figure 7). As mentioned previously, we applied some compensatory rates (for K*c1.1*–K*c1.4*, 12.50, 0.10, 37.4 and 0.50, respectively) in this scenario to render the molecular flows more physiological and the results more accurate. After compensation, we re–validated the system (Table 1), and almost all the estimated values (except for IsoNeuroPs and Vit E) were within the 95% CI. This scenario showed about 13–fold more PUFA than the others, and it also showed higher ROS, whereas Vit E was quite similar. Supra–nutritional PUFA resulted in an almost 3–fold higher level of IsoNeuroPs and Aldehydes compared with the standard model.

## 4. Discussion

Organisms exhibit a myriad of homeostatic mechanisms based on internal and external interactions, which modulate biological responses at different scales and dynamical rates [61]. Building a mathematical model can be an effective way to describe and understand some aspects of such variability. The best outcome of such models is to predict the behaviour of these complex systems, returning values close to the measured one. The rationale of modelling is based on the simplification of the system by assuming some approximations and uncertainty. Moreover, to better adhere to in vivo values, the model should approximate the complexity and the number of variables of the biological system as much as possible. According to such methodological needs, in the present study we proposed a modelling strategy for a living system, using as a case study the metabolism of fat–soluble nutrients in the rabbit. Within this framework, the dynamic model outlined some crucial aspects in the balance of the ROS–PUFA–antioxidant system.

In this model system, we evaluated some common OUTPUTs that approximate the oxidative status of the investigated organism, although the quantification of some of them (i.e., ROS, aldehydes, isoprostanoids) is not fully representative of oxidative stress and requires mentioning their specific chemical entities [62]. Starting from a standard condition (standard diet), we introduced dietary changes in the model system (VIT E+ and PUFA+ scenarios) and predicted the relative OUTPUTs (ROS, PUFA, Vit E, AA, IsoNeuroPs, Aldehydes and Vit Eox). We estimated the accuracy of the prediction by comparing analytical data obtained in the corresponding experimental models of nutritional intervention (Figure 4, Table 1). The accuracy of the model was high and the OUTPUT variables were well described by the simulation in both the standard and VIT E+ scenarios, but not in the PUFA+ scenario.

VIT E+ resulted in IsoNeuroP and AA levels similar to the standard model; on the contrary, the Aldehyde levels were reduced. Vit E levels were increased slightly, but its oxidised form (Vit Eox) was 3–fold higher in this comparison. As expected, the addition of 200 mg/kg of α–tocopheryl acetate (VIT E+) reduced the global oxidation index (Aldehydes) of the standard model without affecting specific oxidation markers (IsoNeuroPs). Although excess Vit E may lead to undesirable effects, such as accumulation of this vitamin in fat depots [20] and its increased exposure to autoxidation processes [24,29], based on the formation of α–TQ, the system can utilise a certain amount of the administered Vit E to donate H atoms to other substrates. This finding confirms its redox interaction with the other lipids of the system. In fact, the Vit E levels remained in the physiological range [24] in VIT E+ conditions, that is, similar to the levels observed in animals fed a standard diet (458.41 × 10^−5^ vs. 422.00 × 10^−5^ moles, respectively).

The PUFA+ scenario presented a marked increase in blood PUFA (about 13 times higher than the other scenarios). This elevation apparently destabilised the main relationship between key molecules involved in detoxification of PUFA–derived oxidative products (i.e., Vit E and its oxidised form). Accordingly, when PUFA INPUT was >55.5 moles (e.g., 80 moles), the model became unstable (Figure 6) and the outcomes lacked accuracy. Hence, we introduced compensatory rates in the model. This type of correction is not a mathematical artifact due to fitting analytical and estimated values, but rather a way to keep considering metabolic changes that occur in a system that becomes unstable.

It is widely known that high dietary levels of PUFA increase the risk of autoxidation and could impair the antioxidant defence of the organism [12,63]. Moreover, deficient antioxidant defence in a tissue could result in higher risk of chronic inflammation and development of degenerative diseases [64]. Obviously, increased PUFA intake was well tolerated in the healthy rabbits utilised as the experimental model in this study. This elevated intake stimulated compensatory responses relevant to the redox homeostasis of the animal. Considering these facts, we introduced different compensatory rates (K*c1*–K*c4* in the PUFA+ scenario. These compensatory rates represent critical checkpoints of the system; when self–adjustments are required (e.g., change of flow or genetic reprogramming), this indicates unbalanced homeostasis and the need for appropriate corrections to be introduced in the system (i.e., changes in Vit E/PUFA intake or the reduced glutathione [GSH]/oxidised glutathione [GSSG] ratio, etc.). The reasons for each compensatory factor manually introduced in the system are as follows:

K*c1* aims to counteract isoprostanoid accumulation that is activated when PUFAs concentrations exceed physiological levels. Indeed, in healthy conditions, isoprostanoids circulate in the plasma and are excreted in the urine [27]. However, when these metabolites reach certain levels, they undergo rearrangement, dehydration or conjugation with glucuronide in the liver to yield a variety of secondary metabolites, thus leading to a reduction in their concentrations and the risk of adverse effects [6].

K*c2* compensates for the accumulation of aldehydes in the system. This increases the conversion of 4–hydroxynonenal (4HNE) to less reactive chemical species [20] through three main catabolic reactions: (1) the formation of adducts with GSH, which occurs spontaneously or catalysed by glutathione–S–transferases (GSTs); (2) its reduction to 1,4–dihydroxy–2–nonene (DHN) by aldo–keto reductases (AKRs) or alcohol dehydrogenases (ADH); and 3) its oxidation to 4–hydroxy–2–nonenoic acid (HNA) by aldehyde dehydrogenase (ALDH) [65].

K*c3* intervenes when the levels of the free radical–derived metabolites of Vit E, α–TQ or the same Vit E, are too high (Figure 6), implying increased expression of detoxifying genes encoding CYP450s involved in the metabolism of this vitamin and other antioxidants, as well as of xenobiotics, electrophiles, heavy metals, etc. [66].

Finally, K*c4* compensates for external perturbation variables (i.e., diet), eventually interfering with antioxidant capacity and with oxidative processes.

Independently of these compensations, the PUFA+ scenario showed more PUFA and ROS, whereas Vit E was quite similar to the other models (*p* < 0.05). However, IsoNeuroPs, Aldehydes and Vit E ox were almost 2–3–fold higher compared with the standard model (Table 1). Although the introduction of these compensatory rates improved the accuracy of the PUFA+ scenario, some traits (Vit E, IsoNeuroPs) remained slightly outside the 95% CI. The differences observed could be ascribed to different factors: on the one hand, the sensitivity of analytical methods, including systematic errors of the measurements, are not considered in the model; on the other hand, the model has limits because it forcefully misses or miscalculates some molecular interactions and their outcomes. Furthermore, the accuracy of the model should improve when the number of samples increases.

In this view, comparison of in vivo data with what is estimated by the model highlights some advantages and disadvantages of the modelling strategy. Regarding advantages, the model provides an integrated tool to assess the time–course of a complex set of variables representing a comprehensive and dynamic picture of the system. This information cannot be obtained from punctual analyses and techniques that describe the system only with static snapshots. Regarding disadvantages, the model is naturally based on limitations and assumptions dealing with the complexity of the aspects considered in the study. Furthermore, some of them are still unknown or undetermined.

There are only a few examples in the literature of similar procedures for modelling biological processes. Musesti et al. [67] and Giantesio et al. [68] created a mathematical model of muscle tissue modification to study cell ageing in sarcopenia, using physiological parameters (i.e., muscle mass, electrical impedance) and variations in external factors (e.g., diet, physical activity, pharmacological treatments, environmental pollution exposure, etc.). They applied the model to analyse two typical symptoms of sarcopenia [68]: loss of mass and loss of force, finding non–linear functions that described these alterations of muscle physiology. These authors also applied a compensatory strategy. Specifically, they developed these functions by defining some constants (identified in their studies as *d*) representing the constant damage associated with the different symptoms of sarcopenia. This approach demonstrated the relevance of modulating these constants in the simulation to depict different scenarios of the biological system.

Although our approach may have biological and mathematical limits, it is apparent that it could be very useful when applied to determine single or combined effects of dietary interventions on the redox homeostasis of the rabbit. In particular, the scientific and technical literature has shown that rabbit bucks and/or does require high levels of PUFA and Vit E for suitable reproductive performance [53,69]. Indeed, germ cells have a particularly high level of LC–PUFA in their membranes. Thus, to ensure good fertility, it is necessary to include certain amounts of PUFA (i.e., LA, ALA, EPA, DPA, and DHA) and antioxidants (i.e., Vit E and AA) in their diets [14]. However, the levels of these compounds should be regulated objectively and carefully; otherwise, they could miss or even hinder the expected improvements. For these reasons, the development of modelling tools holds great potential for precision nutrition studies using animal models and humans.

## 5. Conclusions

We developed a mathematical model based on computer software that mimics the complex interactions of the ROS–PUFA–antioxidant system in rabbit, namely the VENSIM model. This can be used to estimate *a priori* the effect of dietary treatments (i.e., antioxidants, PUFAs) on the redox homeostasis of the rabbit, and in turn on the animal’s health.

In this view, the model can assist in assessing the effect of oxidative challenges and dietary interventions with bioactive lipids such as Vit E and PUFA on the reproductive efficiency of animals/humans, which is a physiological function very sensitive to redox imbalance and oxidative stress. This model system can be implemented and easily be instructed to simulate a wide panel of processes and to assess several phenotypic traits.

In particular, self–learning processes able to identify potential health risks linked to imbalances of the system can be developed. Further improvements of the model would permit studying other systems (i.e., male/female reproduction, key organs, etc.) and the response to exogenous factors different from diets, such as environmental, pathological and pharmacological perturbations.

## Figures and Tables

**Table 1:** Analytic (mean ± SE and lower/upper confidence intervals) and VENSIM resulted values (moles) of OUTPUTs used for calibration and validation of models (standard, VIT E+ and PUFA+).

VENSIM INPUT ^1^	OUTPUT ^2^	Mean ± SE (Analytic Determination)	Confidence Interval (95%)		VENSIM Values
			Lower Limit	Upper Limit	
**standard model**					
7000	ROS	5704.29 ± 101.53 a	5474.59	5933.99	5600.00
5.10	PUFA	5.27 ± 0.08 a	5.01	5.54	5.48
0.0024	Vit E	422.00 ± 6.95 × 10^−5^ a	400.61 × 10^−5^	435.60 × 10^−5^	401.00 × 10^−5^
0.0026	AA	929.74 ± 34.66 × 10^−5^	851.80 × 10^−5^	1008.67 × 10^−5^	920.00 × 10^−5^
–	IsoNeuroPs	0.1494 ± 0.026 a	0.1434	0.1554	0.1400
–	Aldehydes	10.10 ± 0.16 a	9.72	10.48	9.00
–	Vit Eox	5.99 ± 0.69 × 10^−5^ a	4.63 × 10^−5^	7.33 × 10^−5^	4.98 × 10^−5^
**VIT E +**					
7000	ROS	6690.82 ± 164.04 b	6319.71	7061.93	6720.00
5.10	PUFA	6.62 ± 0.50 a	5.49	7.75	5.50
0.0030	Vit E	458.41 ± 22.2 × 10^−5^ a	408.82 × 10^−5^	501.00 × 10^−5^	457.00 × 10^−5^
0.0026	AA	937.48 ± 47.60 × 10^−5^	829.93 × 10^−5^	1045.73 × 10^−5^	928.00 × 10^−5^
–	IsoNeuroPs	0.1412 ± 0.00329 a	0.1338	0.1487	0.140
–	Aldehydes	7.45 ± 0.27 b	6.84	8.07	7.02
–	Vit Eox	16.42 ± 1.44 × 10^−5^ b	11.71 × 10^−5^	20.50 × 10^−5^	12.60 × 10^−5^
**PUFA +**					
7000	ROS	7871.43 ± 227.15 b	7357.57	8385.30	7416.00
600	PUFA	82.15 ± 3.58 b	74.17	90.12	76.99
0.0030	Vit E	260.22 ± 8.75 × 10^−5^ b	243.06 × 10^−5^	277.38 × 10^−5^	**298.00 × 10** **^−5^**
0.0026	AA	976.78 ± 31.99 × 10^−5^	904.46 × 10^−5^	1049.50 × 10^−5^	953.30 × 10^−5^
–	IsoNeuroPs	0.2566 ± 0.0608 b	0.2412	0.2720	**0.3000**
–	Aldehydes	14.05 ± 0.40 c	13.13	14.97	14.81
–	Vit Eox	4.67 ± 0.77 × 10^−5^ a	2.92 × 10^−5^	6.19 × 10^−5^	4.69 × 10^−5^

Different letters (a–c) for the same traits in different scenarios mean *p* < 0.05. ^1^ INPUT represents the starting values introduced in the VENSIM model (moles), in order to calculate the OUTPUTs. ^2^ ROS, reactive oxygen species; PUFA, polyunsaturated fatty acids; vit E, vitamin E; AA, ascorbic acid; IsoNeuroPs, iso– and neuro–prostanes; Vit Eox, oxidized vitamin E.

## Data Availability

Data is contained within the article.

## Appendix A

**Table A1:** Formulation, proximate analysis and fatty acid profile of the control (Standard) and polyunsaturated + (PUFA+) and vitamin E + diet (Vit E+).

	Units	Standard	VIT E+	PUFA +
Ingredients				
Dehydrated alfalfa meal	g/kg	300	300	380
Soybean meal 44%	g/kg	150	150	100
Barley meal	g/kg	410	410	310
Wheat bran	g/kg	52	52	52
Soybean oil	g/kg	30	30	–
Extruded flaxseed	g/kg	–	–	100
Beet molasses	g/kg	20	20	10
Calcium carbonate	g/kg	7	7	7
Calcium diphosphate	g/kg	13.5	13.5	13.5
Salt	g/kg	7	7	7
DL–methionine	g/kg	0.5	0.5	0.5
Vitamin–mineral premix ^1^	g/kg	10	10	10
α–tocopheryl acetate	mg/kg	–	200	200
Proximate composition				
Crude protein	g/kg	174	174	174
Ether extract	g/kg	47.7	47.7	47.2
Crude fibre	g/kg	122	122	137
Ash	g/kg	89	89	84
Fatty acids provided by flaxseed				
C18:3 ALA	g/kg	9.23	9.05	12.08

^1^ Per kg diet: vitamin A, 11,000 IU; vitamin D3, 2000 IU; vitamin B1, 2.5 mg; vitamin B2, 4 mg; vitamin B6, 1.25 mg; vitamin B12, 0.01 mg; alpha–tocopheryl acetate, 5 mg; biotin, 0.06 mg; vitamin K, 2.5 mg; niacin, 15 mg; folic acid, 0.30 mg; d–pantothenic acid, 10 mg; choline, 600 mg; Mn, 60 mg; Fe, 50 mg; Zn, 15 mg; I, 0.5 mg; Co, 0.5 mg. – indicates that the specific ingredient was not included in the diet. [52].

## References

[1] Murphy M.P., Holmgren A., Larsson N.G., Halliwell B., Chang C.J., Kalyanaraman B., Rhee S.G., Thornalley P.J., Partridge L., Gems D. (2011). Unraveling the biological roles of reactive oxygen species. Cell Metab..

[2] Lauridsen C. (2019). From oxidative stress to inflammation: Redox balance and immune system. Poult. Sci..

[3] Chance B., Sies H., Boveris A. (1979). Hydroperoxide metabolism in mammalian organs. Physiol. Rev..

[4] Reeg S., Grune T. (2015). Protein Oxidation in Aging: Does It Play a Role in Aging Progression?. Antioxid. Redox Signal..

[5] Kanvah S., Joseph J., Schuster G.B., Barnett R.N., Cleveland C.L., Landman U.Z.I. (2010). Oxidation of DNA: Damage to nucleobases. Acc. Chem. Res..

[6] Milne G.L., Dai Q., Roberts L.J. (2015). The isoprostanes-25 years later. Biochim. Biophys. Acta-Mol. Cell Biol. Lipids.

[7] Ali S.S., Ahsan H., Zia M.K., Siddiqui T., Khan F.H. (2020). Understanding oxidants and antioxidants: Classical team with new players. J. Food Biochem..

[8] Finkel T., Holbrook N.J. (2000). Oxidants, oxidative stress and the biology of ageing. Nature.

[9] Agarwal A., Virk G., Ong C., du Plessis S.S. (2014). Effect of Oxidative Stress on Male Reproduction. World J. Mens. Health.

[10] Halliwell B. (1999). Antioxidant defence mechanisms: From the beginning to the end (of the beginning). Free Radic. Res..

[11] Sies H. (1999). Glutathione and its role in cellular functions. Free Radic. Biol. Med..

[12] Mattioli S., Collodel G., Signorini C., Cotozzolo E., Noto D., Cerretani D., Micheli L., Fiaschi A.I., Brecchia G., Menchetti L. (2021). Tissue antioxidant status and lipid peroxidation are related to dietary intake of n-3 polyunsaturated acids: A rabbit model. Antioxidants.

[13] Griveau J.F., Le Lannou D. (1997). Reactive oxygen species and human spermatozoa: Physiology and pathology. Int. J. Androl..

[14] Castellini C., Mattioli S., Signorini C., Cotozzolo E., Noto D., Moretti E., Brecchia G., Dal Bosco A., Belmonte G., Durand T. (2019). Effect of Dietary n-3 Source on Rabbit Male Reproduction. Oxid. Med. Cell. Longev..

[15] Foreman J., Demidchik V., Bothwell J.H.F., Mylona P., Miedema H., Angel Torres M., Linstead P., Costa S., Brownlee C., Jones J.D.G. (2003). Reactive oxygen species produced by NADPH oxidase regulate plant cell growth. Nature.

[16] Sauer H., Rahimi G., Hescheler J., Wartenberg M. (2000). Role of reactive oxygen species and phosphatidylinositol 3-kinase in cardiomyocyte differentiation of embryonic stem cells. FEBS Lett..

[17] Niethammer P., Grabher C., Look A.T., Mitchison T.J. (2009). A tissue-scale gradient of hydrogen peroxide mediates rapid wound detection in zebrafish. Nature.

[18] Chakrabarti S., Visweswariah S.S. (2020). Intramacrophage ROS Primes the Innate Immune System via JAK/STAT and Toll Activation. Cell Rep..

[19] Foyer C.H., Noctor G. (2005). Redox homeostasis and antioxidant signaling: A metabolic interface between stress perception and physiological responses. Plant Cell.

[20] Torquato P., Bartolini D., Giusepponi D., Piroddi M., Sebastiani B., Saluti G., Galarini R., Galli F. (2019). Increased plasma levels of the lipoperoxyl radical-derived vitamin E metabolite α-tocopheryl quinone are an early indicator of lipotoxicity in fatty liver subjects. Free Radic. Biol. Med..

[21] Suburu J., Gu Z., Chen H., Chen W., Zhang H., Chen Y.Q. (2013). Fatty acid metabolism: Implications for diet, genetic variation, and disease. Food Biosci..

[22] Simopoulos A.P. (2000). Human requirement for N-3 polyunsaturated fatty acids. Poult. Sci..

[23] Simopoulos A.P. (2002). Genetic variation and dietary response: Nutrigenetics/nutrigenomics. Asia Pac. J. Clin. Nutr..

[24] Castellini C., Mourvaki E., Dal Bosco A., Galli F. (2007). Vitamin E biochemistry and function: A case study in male rabbit. Reprod. Domest. Anim..

[25] Mattioli S., Dal Bosco A., Duarte J.M.M., D’Amato R., Castellini C., Beone G.M., Fontanella M.C., Beghelli D., Regni L., Businelli D. (2019). Use of Selenium-enriched olive leaves in the feed of growing rabbits: Effect on oxidative status, mineral profile and Selenium speciation of Longissimus dorsi meat. J. Trace Elem. Med. Biol..

[26] Signorini C., Cardile V., Pannuzzo G., Graziano A.C.E., Durand T., Galano J.M., Oger C., Leoncini S., Cortelazzo A., Lee J.C.Y. (2019). Increased isoprostanoid levels in brain from murine model of Krabbe disease–Relevance of isoprostanes, dihomo-isoprostanes and neuroprostanes to disease severity. Free Radic. Biol. Med..

[27] Ahmed O., Galano J.-M., Pavlickova T., Revol-Cavalier J., Vigor C., Lee J.-Y., Oger C., Durand T. (2020). Moving forward with isoprostanes, neuroprostanes and phytoprostanes: Where are we now?. Essays Biochem..

[28] Signorini C., Moretti E., Noto D., Mattioli S., Castellini C., Pascarelli N.A., Durand T., Oger C., Galano J.M., De Felice C. (2021). F4-neuroprostanes: A role in sperm capacitation. Life.

[29] Galli F., Azzi A., Birringer M., Cook-Mills J.M., Eggersdorfer M., Frank J., Cruciani G., Lorkowski S., Özer N.K. (2017). Vitamin E: Emerging aspects and new directions. Free Radic. Biol. Med..

[30] Traber M.G., Atkinson J. (2007). Vitamin E, antioxidant and nothing more. Free Radic. Biol. Med..

[31] Torquato P., Giusepponi D., Galarini R., Bartolini D., Piroddi M., Galli F. (2019). Analysis of vitamin E metabolites. Vitamin E: Chemistry and Nutritional Benefits.

[32] Meunier B., de Visser S.P., Shaik S. (2004). Mechanism of oxidation reactions catalyzed by cytochrome P450 enzymes. Chem. Rev..

[33] Sies H., Ursini F. (2022). Homeostatic control of redox status and health. IUBMB Life.

[34] Xiong Y.L. (2000). Protein Oxidation and Implications for Muscle Food Quality. Antioxidants in Muscle Foods: Nutritional Strategies to Improve Quality.

[35] Xiong J. (2018). Fatty Acid Oxidation in Cell Fate Determination. Trends Biochem. Sci..

[36] Suckow M.A., Stevens K.A., Wilson R.P. (2012). The Laboratory Rabbit, Guinea Pig, Hamster, and Other Rodents.

[37] Sargent R.G. (2010). Verification and validation of simulation models. Proc.-Winter Simul. Conf..

[38] Trucano T.G., Swiler L.P., Igusa T., Oberkampf W.L., Pilch M. (2006). Calibration, validation, and sensitivity analysis: What’s what. Reliab. Eng. Syst. Saf..

[39] Dimokas G., Tchamitchian M., Kittas C. (2009). Calibration and validation of a biological model to simulate the development and production of tomatoes in Mediterranean greenhouses during winter period. Biosyst. Eng..

[40] VENSIM, Vensim(R) PLE, Version 9.0.1, Ventana Systems, Inc. https://vensim.com/vensim-personal-learning-edition.

[41] Brieger K., Schiavone S., Miller F.J., Krause K.H. (2012). Reactive oxygen species: From health to disease. Swiss Med. Wkly..

[42] Turchini G.M., Francis D.S., De Silva S.S. (2007). A whole body, in vivo, fatty acid balance method to quantify PUFA metabolism (desaturation, elongation and beta-oxidation). Lipids.

[43] Bargut T.C.L., Frantz E.D.C., Mandarim-De-Lacerda C.A., Aguila M.B. (2014). Effects of a diet rich in n-3 polyunsaturated fatty acids on hepatic lipogenesis and beta-oxidation in mice. Lipids.

[44] Mouchlis V.D., Hayashi D., Vasquez A.M., Cao J., McCammon J.A., Dennis E.A. (2022). Lipoprotein-associated phospholipase A2: A paradigm for allosteric regulation by membranes. Proc. Natl. Acad. Sci. USA.

[45] Min B., Ahn D.U. (2005). Mechanism of lipid peroxidation in meat and meat products-A review. Food Sci. Biotechnol..

[46] Fereidoon S., Ying Z. (2010). Lipid oxidation and improving the oxidative stability. Chem. Soc. Rev..

[47] Galli F., Polidori M.C., Stahl W., Mecocci P., Kelly F.J. (2007). Vitamin E biotransformation in humans. Vitam. Horm..

[48] Torquato P., Giusepponi D., Bartolini D., Barola C., Marinelli R., Sebastiani B., Galarini R., Galli F. (2021). Pre-analytical monitoring and protection of oxidizable lipids in human plasma (vitamin E and ω−3 and ω−6 fatty acids): An update for redox-lipidomics methods. Free Radic. Biol. Med..

[49] Meister A. (1994). Glutathione-ascorbic acid antioxidant system in animals. J. Biol. Chem..

[50] Combs G.F. (2015). Biomarkers of selenium status. Nutrients.

[51] De Blas C., Wisewan J. (2020). Nutrition of the Rabbit.

[52] Castellini C., Lattaioli P., Dal Bosco A., Beghelli D. (2002). Effect of supranutritional level of dietary α-tocopheryl acetate and selenium on rabbit semen. Theriogenology.

[53] Rodríguez M., Rebollar P.G., Mattioli S., Castellini C. (2019). n-3 PUFA sources (precursor/products): A review of current knowledge on rabbit. Animals.

[54] Folch J., Lees M., Sloane Stanley G.H. (1957). A simple method for the isolation and purification of total lipides from animal tissues. J. Biol. Chem..

[55] Christie W.W. (1982). A simple procedure for rapid transmethylation of glycerolipids and cholesteryl esters. J. Lipid Res..

[56] Mattioli S., Machado Duarte J.M., Castellini C., D’Amato R., Regni L., Proietti P., Businelli D., Cotozzolo E., Rodrigues M., Dal Bosco A. (2018). Use of olive leaves (whether or not fortified with sodium selenate) in rabbit feeding: Effect on performance, carcass and meat characteristics, and estimated indexes of fatty acid metabolism. Meat Sci..

[57] Ross M.A. (1994). Determination of ascorbic acid and uric acid in plasma by high-performance liquid chromatography. J. Chromatogr. B Biomed. Sci. Appl..

[58] Shara M.A., Dickson P.H., Bagchi D., Stohs S.J. (1992). Excretion of formaldehyde, malondialdehyde, acetaldehyde and acetone in the urine of rats in response to 2,3,7,8-tetrachlorodibenzo-p-dioxin, paraquat, endrin and carbon tetrachloride. J. Chromatogr. B Biomed. Sci. Appl..

[59] Longini M., Moretti E., Signorini C., Noto D., Iacoponi F., Collodel G. (2020). Relevance of seminal F2-dihomo-IsoPs, F2-IsoPs and F4-NeuroPs in idiopathic infertility and varicocele. Prostaglandins Other Lipid Mediat..

[60] SAS (2015). Proc GLM in SAS, Release 9.4.

[61] Feature T. (2011). A Living System By Monya Baker. Nature.

[62] Sies H. (2015). Oxidative stress: A concept in redox biology and medicine. Redox Biol..

[63] Bartolini D., Torquato P., Barola C., Russo A., Rychlicki C., Giusepponi D., Bellezza G., Sidoni A., Galarini R., Svegliati-Baroni G. (2017). Nonalcoholic fatty liver disease impairs the cytochrome P-450-dependent metabolism of α-tocopherol (vitamin E). J. Nutr. Biochem..

[64] Chakraborty P., Dugmonits K.N., Végh A.G., Hollandi R., Horváth P., Maléth J., Hegyi P., Németh G., Hermesz E. (2019). Failure in the compensatory mechanism in red blood cells due to sustained smoking during pregnancy. Chem. Biol. Interact..

[65] Zhong H., Yin H. (2015). Role of lipid peroxidation derived 4-hydroxynonenal (4-HNE) in cancer: Focusing on mitochondria. Redox Biol..

[66] Bartolini D., Marinelli R., Giusepponi D., Galarini R., Barola C., Stabile A.M., Sebastiani B., Paoletti F., Betti M., Rende M. (2021). Alpha-tocopherol metabolites (the vitamin E metabolome) and their interindividual variability during supplementation. Antioxidants.

[67] Musesti A., Giusteri G.G., Marzocchi A. (2014). Predicting ageing: On the mathematical modelization of ageing muscle tissue. 821 Active Ageing and Healthy Living.

[68] Giantesio G., Marzocchi A., Musesti A. (2018). Loss of mass and performance in skeletal muscle tissue: A continuum model. Commun. Appl. Ind. Math..

[69] Mattioli S., Dal Bosco A., Maranesi M., Petrucci L., Rebollar P.G., Castellini C. (2019). Dietary fish oil and flaxseed for rabbit does: Fatty acids distribution and Δ6-desaturase enzyme expression of different tissues. Animal.

